# Supplementary material for: The prevalence, incidence, and risk factors of mental health problems and mental health service use before and 9 months after the COVID-19 outbreak among the general Dutch population. A 3-wave prospective study
Source: PLoS One. 2022 Nov 10;17(11):e0276834. doi: 10.1371/journal.pone.0276834 (PMC9648763; doi:10.1371/journal.pone.0276834)
Supplement: Attachment — Submitted filename: letter to reviewers 19-8-2022.docx [file pone.0276834.s003.docx]

We thank the reviewers very much for their time and effort. It certainly helped us to improve our manuscript.

**Reviewer #1:**

1. This paper is improved from my initial reading and the decision to rewrite the introduction means it now makes much more sense. However, there remain some flaws in the work, which could be addressed.

**Response:**

Thank you for your kind words.

2. Place the last paragraph on pp. 8-9 before the aim of the study so it flows neatly.

**Response:**

We have moved this section as suggested and renumbered the references.

3. Ethical approval/informed consent p. 10; it would probably be wise to give the reason why ethics was not needed; aggregated anonymised data. Agree with mentioning that participants had agreed for further analysis of their data.

**Response:**

Based on this comment, we have revised the first sentence as follows:

*“Since our research did not impose certain (experimental) behavior, our research did not need the approval of a Dutch Medical Ethical Testing committee according to the Dutch Law (see https://english.ccmo.nl/investigators/legal-framework-for-medical-scientific-research/your-research-is-it-subject-to-the-wmo-or-not).”*

4. p.11, p. 16, Table 2, Table 3 etc.,: ‘disabilities due to mental health problems’. This could perhaps be worded as reduced or impaired functioning and use the ICF language. Disability using the social model refers to the barriers within society, used otherwise it places the ‘problem’ within the individual and makes the paper biomedical and lacking in consideration for social barriers. This makes people the problem. Means-Christensen et al. do not use the term disability in relation to psychological distress, they use impaired functioning (p. 566) which is the language of the ICF, as such the article is somewhat misrepresented.

**Response:**

Based on this comment and to prevent possible confusion we have replaced “disabilities” with “impaired functioning” throughout the manuscript, including the tables and appendix 1 and 2.

5. Likewise, reword mental health problems throughout the paper to mental health difficulties. Language is extremely important so we do not reify and marginalise groups.

**Response:**

We fully agree with the reviewer that language is very important. However, we do not understand that the term “mental health problems” marginalizes groups. In addition, a search on PUBMED with the term “mental health problems” identified 16,461 papers while as search with the term "mental health difficulties" identified “only” 1,226 papers, clearly suggesting that “mental health problems” is an often used and well accepted used term (we used this term in all our papers). For these reasons we did not reword the term “mental health problems”.

6. The term ‘eating problems’ could be amended. I thought these were eating disorders, are these identified by type in the LISS data? Then sleep problems are not identified, this could be a critique of the LISS data collection, does it mean disturbance in circadian rhythms, insomnia, or does it mean sleep disorders such as breathing-related sleep disorders? Alternatively, are a number of conditions/disorders aggregated under one heading? A little more clarity and critique here about the data would open the study out.

**Response:**

Based on this comment we have replaced “eating problems” with “eating disorders”. In addition, we added:

*“As described, sleep problems, fatigue and impaired functioning were assessed by one item questions. Future research is needed to examine the course of different aspects of sleep problems, fatigue and impaired functioning”*.

7. p.22: 2nd sentence, should it not be that existing mental health difficulties and service use were the strongest predictor for continued mental health difficulties and service use before and after the COVID-19 pandemic?

**Response:**

We understand the comment of the reviewer, but to be able to state that existing mental health difficulties and service use were the strongest predictor for continued mental health difficulties and service use before and after the COVID-19 pandemic, different analyses are required. In this case the dependent variables should be persistent mental health problems and service use.

8. Services use should be service use throughout the paper, or use of services. Appreciate this a language issue.

**Response:**

We have revised the text using the term “service use”.

9. p.29, 2nd paragraph: ‘people who ran into mental health issues’ people experience mental health difficulties, they do not ‘run into them’ please amend.

**Response:**

Thank you for noticing this error. We have corrected this mistake.

10. Limitations: this section goes further than strengths and limitations, so place some of this in a conclusion.

**Response:**

Based on this comment we moved the sentence “Monitoring of the mental health of the general population is needed because the duration of this pandemic on the longer term may undermine the capacity of individuals to cope with the consequences” to the final conclusions section.

11. Final remarks section: I am not convinced of the rationale in the comments in this section because I feel it adds little to the paper. Offering differences between the UK, Netherlands and USA (observed from other studies, not analysed statistically) was not the aim of the paper and adding these last six lines detracts from the work done. The conclusion needs to focus on the paper, summing it up and its addition/contribution to existing research. I would delete this section and rewrite. Perhaps call it conclusion rather than final remarks begin with prospective studies and use some of the limitation section, which is more suited to a conclusion section. For example, the results suggesting further research using clinical interviews to provide more insight and future studies on mental health difficulties, eating disorders etc.

**Response:**

Based on this comment we replaced “final remarks” with “final conclusions”.

We are sorry, but we disagree with reviewer’s comments on this section. It is the task of researchers to compare the findings of other researchers with their findings. This is what we did in the discussion section. We therefore do not understand reviewers remark “*Offering differences between the UK, Netherlands and USA (observed from other studies, not analysed statistically) was not the aim of the paper ……”.* In addition, in line with the comments of the reviewer we stated “*Future comparative multi-country studies, including middle and low-income countries, are needed to further disentangle the complex relationships between especially existing welfare and health care systems, the size and dosage of governmental financial support programs, political systems and tensions, preventive measures, and mental health among the general population”*.

**Reviewer #4:**

Dear authors,

12. I consider the comments raised by the reviewers in the previous round of reviews were successfully addressed. Congratulations for your effort on that.

**Response:**

Thank you for your compliments.
